# Reviewed article: Oliveira MB, Oliveira VHFP, Blumenberg C, Silva DT, Herval AM, Paranhos LR. Analysis of health funding and expenditure in the municipalities of Sergipe state, Brazil: an ecological study, 2005-2020. Epidemiol Serv Saude. 2025:34;e20240107

**DOI:** 10.1590/S2237-96222025v34e20240107.a

**Published:** 2025-05-12

**Authors:** Cristiano Bertolossi Marta

**Affiliations:** Universidade do Estado do Rio de Janeiro, Fundamentos de Enfermagem

**Table:** 

Rating	Excellent	Good	Average	Below average	Poor
Interest	✓				
Quality	✓				
Originality	✓				
Overall	✓				

**Table:** 

Questionnaire	Yes	No	Not applicable
Does the manuscript contain new and significant information to justify publication?	✓		
Does the Abstract (Summary) clearly and accurately describe the content of the article?	✓		
Is the problem significant and concisely stated?	✓		
Are the methods described comprehensively?	✓		
Are the interpretations and conclusions justified by the results?	✓		
Is adequate reference made to other work in the field?	✓		

## Manuscript Structure:

Length of article is: Adequate

Number of tables is: Adequate

Number of figures is: Adequate

Please state any conflict(s) of interest that you have in relation to the review of this paper (state “none” if this is not applicable): None

